# Burden of disease of COVID-19 in the department of Nariño, Colombia, 2020-2021

**DOI:** 10.17843/rpmesp.2022.393.10947

**Published:** 2022-09-30

**Authors:** Arsenio Hidalgo-Troya, Jorge Martín Rodríguez, Anderson Rocha-Buelvas, Diana Urrego-Ricaurte

**Affiliations:** 1 Universidad de Nariño, Pasto-Nariño, Colombia. Universidad Antonio Nariño Universidad de Nariño Pasto-Nariño Colombia; 2 Pontificia Universidad Javeriana, Bogotá D.C., Colombia. Pontificia Universidad Javeriana Pontificia Universidad Javeriana Bogotá D.C. Colombia

**Keywords:** SARS-CoV-2, COVID-19, Burden Disease, Disability-adjusted Life Year, Coronavirus, Colombia

## Abstract

**Objectives.:**

To estimate the burden of disease of COVID-19 in the department of Nariño, Colombia, based on the disability-adjusted life years (DALYs) between March 2020 and August 2021.

**Materials and methods.:**

The description and characterization of COVID-19 cases reported between March 2020 and August 2021 was made according to age groups, sex, ethnicity, municipalities of residence and subregions of Nariño by using information from the national surveillance system SIVIGILA. Crude and cumulative mortality rates for COVID-19 were estimated for the previously described variables. Years lost due to premature death (YLL) and years lived with disability (YLD) were calculated. Total DALYs were estimated by adding YLL + YLD. These were calculated by sex, ethnicity, age group and subregions of Nariño. Relative risks were estimated from rate ratios (RR) and 95% confidence intervals for the study variables.

**Results.:**

The highest morbidity, mortality and DALY rates occurred between February and September 2021, in men, in those older than 70 years, in the Afro-descendant ethnic minority group and in the Central, Obando and Juanambú subregions. The burden of disease of COVID-19 in Nariño during the study period is attributed to the YLL, which explain more than 97% of it.

**Conclusions.:**

This is one of the first studies on burden of disease at the regional level, carried out in Colombia, that employs a standardized methodology for COVID-19. This measurement would generate estimates that would allow targeting resources in an intersectoral manner, mitigating the damage to specific populations and geographic areas, especially the most vulnerable ones.

## INTRODUCTION

Coronaviruses behave as a zoonosis ^1^ affecting human health in different periods. In the last 20 years they have caused several epidemics. The first was caused by SARS (severe acute respiratory syndrome); between 2002-2003 this strain infected more than 8000 people (China, United States, Canada, among others) with a lethality rate close to 10%. The second one occurred in 2012, by MERS-CoV (Middle East Respiratory Syndrome); this epidemic affected about 857 people with a mortality rate close to 35% ^2^. The third outbreak was produced by a new strain (SARS-CoV-2), which appeared at the end of 2019 ^3^ causing the healthcare emergency due to COVID-19, with lethality rates between 1-10% worldwide ^4^. The high transmissibility of this virus is due to the rapid respiratory dissemination, because viral transmission can occur by symptomatic patients or asymptomatic individuals ^5^.

Although SARS-CoV-2 is a partially preventable and treatable disease, it has been a health, economic, political, and social challenge for the world. For low- and middle-income countries, it has been the worst humanitarian and public health crisis in their history ^6^, with considerable differences in morbidity and mortality rates among them ^7^. Latin America has precarious healthcare systems unable to provide effective responses, with health budgets below 4.0% of the gross domestic product (GDP) ^8^, which limits access to quality healthcare, making them susceptible to collapse. During the pandemic, governments did not allocate economic and social resources efficiently to the most affected regions, which led to worsening of economic and social inequalities ^9^.

There are other social determinants specific to the continent that have an impact on population health. For example, in Colombia, 37% of households do not eat three meals a day, and 43.9% of heads of household perceive that their economic status has worsened ^10^. Colombia has the second highest unemployment rate after Haiti, where women are the most affected (39%) compared to men (18%). Likewise, labor informality and the widening of the social inequality gap in Brazil and Colombia are very high ^11^, but this gap also exists between regions in Colombia ^12^. Some of the aforementioned determinants may have contributed to the fact that in mid-June 2021 the epidemiological outlook for COVID-19 in Colombia was one of the most unfavorable in the world ^13^, affecting people with comorbidities, who required specialized care ^14^.

In Colombia, the first case of COVID-19 was reported in March 2020. By August 2021 SARS-CoV-2 had spread throughout the country with nearly five million confirmed cases and more than 122,000 deaths. By the end of 2020, the department of Nariño recorded 88,927 confirmed cases and 3381 deaths, with a cumulative incidence of 48/1000 inhabitants and mortality of 1.8/1000 inhabitants ^15^.

In low- and middle-income countries, the evaluation of population health status has traditionally been based on mortality indicators such as general or specific mortality rates, infant mortality, maternal mortality ratio, among others; and/or morbidity indicators such as incidence or prevalence, depending on the type of event under study. Due to their limitations in the measurement of the health-disease phenomena, there is a need to develop other indicators to measure related aspects such as the quality of life of people. In this context, burden of disease studies makes it possible to identify various health problems that cause death, morbidity, and disability, since they integrate the burden produced by premature death, duration, sequelae of the disease and disability through the disability-adjusted healthy life years indicator (DALYs) ^16^.

Colombia has several previous burden of disease studies, including those conducted by the Universidad Javeriana ^17^ ^,^ ^18^ and the National Health Observatory ^19^. 

The department of Nariño is the only one in Colombia that has a disease burden study by subregion conducted by its health authority; in part, because it is a border region characterized by jointly possessing: contexts of armed conflict, high levels of corruption and territories with population at and below the poverty line ^12^, which increases the risk of COVID-19 mortality; hence the relevance of studying the COVID-19 situation based on the estimation of departmental ^20^ and, in this case, subregional DALYs for the department of Nariño.

In the current scenario, it is important to conduct not only national but also territorial burden of disease studies, since according to the National Administrative Department of Statistics (DANE) and the National Institute of Health of Colombia (INS), COVID-19 is currently one of the most important causes of mortality and, therefore, it is essential to identify the burden attributed to this event, in order to guide targeted actions and strategies ^21^. The aim of the study was to determine the burden of disease attributed to COVID-19 in the department of Nariño, between March 2020 and August 2021.

**KEY MESSAGES**

Motivation for the study: burden of disease studies are methodological strategies that contribute to the analysis of population health status. This is one of the first studies on COVID-19 that estimates disability-adjusted healthy life years (DALYs), adapting a methodology to a Latin American context.

Main findings: the burden of disease in Nariño, at the beginning of the epidemic, was mainly due to premature mortality, in men, older than 70 years, of undetermined ethnic groups and in territories previously affected by chronic non-communicable diseases.

Implications: the estimation of DALYs guides decision-makers in generating priorities for resource intervention and control/prevention strategies for this emerging disease based on scientific evidence.

## MATERIALS AND METHODS

This is a descriptive analytical study that uses the records of COVID-19 cases reported to the National Public Health Surveillance System (SIVIGILA), which is coordinated by the INS, as the source of information. For this study, we used the records of the department of Nariño.

Based on information from SIVIGILA, we carried out the description and characterization of COVID-19 cases reported from March 2020 to August 2021, by age grouped as follows: (0-4, 5-14, 15-29, 30-44, 45-59, 60-69, 70-79 and 80 and +); sex (men and women); ethnicity (1: Indigenous, 2: Gypsies, 3-5: Afro-descendant, 6: Other ); municipalities (all municipalities in the department of Nariño) and by the thirteen administrative sub-regions managed by the department of Nariño (Obando, Occidente, Centro, Guambuyaco, Juanambú, La Cordillera, La Sabana, Los Abades, Pacífico Sur, Piedemonte Costero, Río Mayo, Sanquianga and Telembí). a We obtained percentage descriptions for each variable. Time was divided into three semesters: March-August 2020 (A), September-February 2021 (B) and March-August 2021 (C).

### Disease burden of COVID-19

Based on DANE 2020 population projections, crude incidence rates of morbidity (contagion) and cumulative mortality by COVID-19 were obtained for the previously described variables (age groups, sex, ethnicity, municipality, and departmental subregion). Statistical significance tests were applied at a level of 0.05 to identify changes in the rate ratios (RR) of DALYs by age, sex, subregion, and period, by means of Wald tests using a generalized linear model.

DALYs were disaggregated by both, years lost due to premature death (YLLD) and years lived due to disability (YLD) using some methodological references as described below. Table 1, which contains the life expectancies (LE) for both sexes, was used to calculate the YLLFs, taking as the main input the lowest mortality rates recorded for each age group in countries with more than 5 million people. The estimate was made by multiplying the LE by the volume of deaths in the same age group, municipality, region, sex, and ethnicity specific to the department of Nariño.

**Table 1:** Life expectancy by age group used for the calculation of years lost due to premature death (YLLD).

Age	LE	Age	LE	Age	LE	Age	LE	Age	LE
0	86.6	22	64.9	44	43.4	66	22.9	88	6.6
1	85.8	23	64.0	45	42.4	67	22.0	89	6.0
2	85.8	24	63.0	46	41.4	68	21.2	90	5.5
3	85.8	25	62.0	47	40.5	69	20.3	91	5.1
4	85.8	26	61.0	48	39.5	70	19.4	92	4.8
5	81.8	27	60.0	49	38.6	71	18.6	93	4.4
6	80.8	28	59.0	50	37.6	72	17.8	94	4.1
7	79.8	29	58.0	51	36.7	73	16.9	95	3.7
8	78.8	30	57.0	52	35.7	74	16.1	96	3.5
9	77.8	31	56.0	53	34.8	75	15.3	97	3.2
10	76.8	32	55.0	54	33.8	76	14.5	98	3.0
11	76.8	33	54.1	55	32.9	77	13.8	99	2.7
12	76.8	34	53.1	56	32.0	78	13.0	100	2.5
13	76.8	35	52.1	57	31.1	79	12.3	101	2.3
14	76.8	36	51.1	58	30.1	80	11.5	102	2.1
15	76.8	37	50.1	59	29.2	81	10.8	103	2.0
16	75.8	38	49.2	60	28.3	82	10.2	104	1.8
17	74.8	39	48.2	61	27.4	83	9.5	105	1.6
18	73.8	40	47.2	62	26.5	84	8.9	106	1.6
19	72.8	41	46.2	63	25.6	85	8.2	107	1.5
20	71.8	42	45.3	64	24.7	86	7.7	108	1.5
21	71.8	43	44.3	65	23.8	87	7.1	109-10	1.4

LE: life expectancy.

Source: Own elaboration based on GBD Technical Training Workshop May 2015.

The equation for calculating the YLLD was as follows:


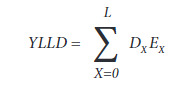


Where: Dx =1 at death at X age; Ex = standard life expectancy of each specific age of the chosen model and L = theoretical limiting age of the chosen life table.

**Figure 1:**
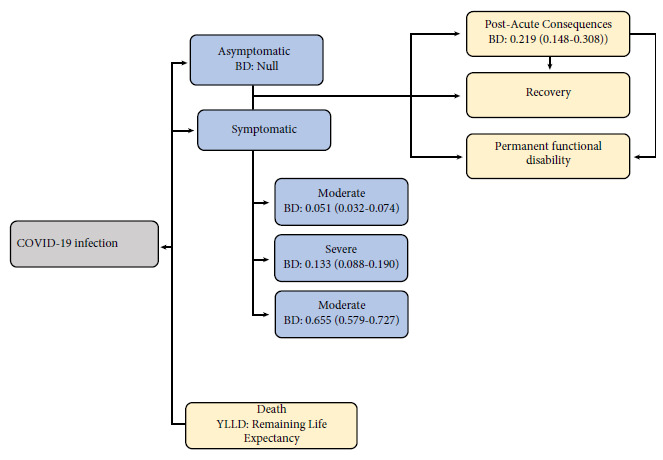
Model for estimating disability-adjusted life-years for COVID-1.

We used the following formula for the estimation of DALYs, or years lived with disability due to COVID-19: *YLD*_*a*_ = D * L. Where: a = the age of onset of the disease, D = burden of the disease, which ranges between 0 and 1. If the individual is completely healthy, a D equal to 0 would be obtained; on the contrary, if the individual dies, D is equal to 1. Severe disease has a value of D close to 1; a mild clinical condition has a value of D close to zero. For this research, regarding the burden of the disease, we used the approach by Wyper *et al*. ^22^, which uses three categories: mild/moderate (0.051), severe (0.133) and critical (0.655) (Figure 1). L=duration of the disease, which, in this case, was estimated as a fraction in years, with the difference between the date of recovery and the date of symptom onset according to SIVIGILA records.

The YLLD and YLD allowed us to calculate the total DALYs by adding them together, as follows: DALYs = YLLD + YLD. Similarly, based on DANE population projections, the total DALY rates per 1000 inhabitants were estimated by sex, ethnicity, age group and subregion in the department of Nariño.

### Negative binomial regression model

Finally, we estimated a generalized linear model to identify the trend in COVID-19 DALY rates for the study period. It was calculated from the number of COVID-19 DALYs, which were taken as a random variable, taking Y = Ln(R), where R represents COVID-19 DALY rates and as independent or explanatory variables, age as X1, sex as X2, time period as X3, and departmental regions as X4. Relative risks were estimated from the rate ratios (RR), together with 95% confidence intervals, for each of the categories of the study variables, based on reference groups for each of the explanatory variables.

For the estimation of the generalized linear model, we used the IBM SPSS version 23 statistical package. The assumptions of equidispersion were verified using Pearson’s deviation and chi-square statistics to establish whether the Poisson regression model was the appropriate model for the data. The goodness-of-fit statistics showed scale values indicating overdispersion, i.e., violation of this property, therefore the alternative negative binomial model was used, and the pseudoR2 statistic was estimated to establish the goodness-of-fit of the model.

### Ethical considerations

For this study, the data were extracted from the records of the National Public Health Surveillance System coordinated by the INS. No modifications were made to the figures provided by the sources of information; whenever reference is made to a text, the authors are cited, while respecting copyright. 

This research is classified as “without risk” according to resolution 8430 of 1993 of the Ministry of Health, in addition, this study has approval certificate No. 004 of April 23, 2020 of the Research Ethics Committee of the Vice-Rectory of Research and Social Interaction of the Universidad de Nariño.

## RESULTS

We found 88,762 cases of COVID-19 infection in the department of Nariño during the period March 2020 and August 2021, with an annual rate of 3196 cases per 100,000 inhabitants. In the same period, deaths from the virus were equal to 2985 cases with an annual mortality rate of 107.5 cases per 100,000 inhabitants.

Regarding the morbidity component, Table 2 shows that the highest cumulative incidence rate (4794 per 100,000 inhabitants) occurred in periods B (1828.3) and C (2121.4). This rate increased gradually with age, the highest rate corresponding to the group aged 80 years and over (7779.8). Women had slightly higher morbidity rates (5034) than men (4556.1). The most affected ethnic minority group were Afro-descendants (4468.3). The most affected subregions were Centro (9955.3) and Juanambú (5400.6), exceeding the departmental average (4794.1). Regarding the mortality component, we observed that the highest cumulative rates occurred in periods B (56.87) and C (69.46). Mortality rates (161.2 per 100,000 population) also increased gradually, with the group aged 80 years and over being the most affected (2367.6). In contrast to morbidity, mortality in men (199.3) was higher than in women (122.9). Once again, the Afro-descendant ethnic group had a higher mortality rate (211). The subregions with the highest mortality from COVID-19 were also Centro (293.6), Obando (180.3) and Juanambú (172.8).

**Table 2:** Cumulative morbidity and mortality due to COVID-19 according to sociodemographic characteristics in the department of Nariño, Colombia, 2020-2021.

Variables	Morbidity			Mortality			p-value
	N	%	Rates (x 100 mil)	N	%	Rates (x 100 mil)	
Time period							< 0.001
A	15,634	17.6	844.4	646	21.6	34.9	
B	33,850	38.1	1828.3	1053	35.3	56.9	
C	39,278	44.3	2121.4	1286	43.1	69.5	
Age							< 0.001
0 - 4	829	0.9	492.9	4	0.1	2.4	
5 - 14	2751	3.1	846.1	2	0.1	0.6	
15 - 29	21,594	24.3	4557.4	35	1.2	7.4	
30 - 44	29,477	33.2	7488.4	221	7.4	56.1	
45 - 59	20,353	22.9	7337.0	595	19.9	214.5	
60 - 69	7371	8.3	6379.0	659	22.1	570.3	
70 - 79	4054	4.6	5982.8	759	25.4	1120.1	
80 or more	2333	2.6	7779.8	710	23.8	2367.6	
Sex							< 0.001
Female	46,417	52.3	5034.0	1133	38.0	122.9	
Male	42,345	47.7	4556.1	1852	62.0	199.3	
Ethnicity							< 0.001
Indigenous	7278	8.2	3525.2	251	8.4	121.6	
Afrodescendant	5936	6.7	4468.2	280	9.4	210.8	
Other	75,548	85.1	4996.0	2454	82.2	162.3	
Subregion							< 0.001
Centro	51,169	57.6	9955.3	1509	50.6	293.6	
Guambuyaco	1371	1.5	3095.5	41	1.4	92.6	
Juanambú	4720	5.3	5400.6	151	5.1	172.8	
La Cordillera	1451	1.6	1754.3	62	2.1	74.9	
La Sabana	2553	2.9	3569.4	107	3.6	149.6	
Los Abades	903	1.0	941.5	35	1.2	36.5	
Obando	12,668	14.3	4124.2	554	18.6	180.4	
Occidente	2183	2.5	4358.9	77	2.6	153.7	
Pacífico Sur	5232	5.9	2189.8	250	8.4	104.6	
Piedemonte Costero	405	0.5	1455.0	32	1.1	114.9	
Río Mayo	4153	4.7	3731.4	101	3.4	90.7	
Sanquianga	917	1.0	728.6	31	1.0	24.6	
Telembí	1037	1.2	1097.3	35	1.2	37.0	
Total	88,762	100.0	4794.1	2985	100.0	161.2	

p-values were calculated using chi-square, which compares mortality and cumulative morbidity.

A: March-August 2020 period, B: September 2020-February 2021, C: March-August 2021 period.

### COVID-19 disease burden

The YLLD were 67,638.6 with an annual rate of 25.3 per thousand inhabitants during the analyzed period. YLD were 313.2 with an annual rate of 0.11 per 1000 inhabitants. DALYs were 97,950.81 with an annual rate of 24.5 per 1000 inhabitants during the evaluated period. Table 3 shows that the third time period had the highest DALYs due to COVID-19 (17.8). By age group, 190 DALYs were lost in those aged 80 years and older; 177.5 DALYs in those aged 70-79 years; 138.61 in those aged 60-69 years and 74.55 in those aged 45-59 years. Men lost 46.3 DALYs, and women lost 27 per 1000 inhabitants. The Afro-descendant ethnic minority group lost 53.2 DALYs. Finally, the subregions with the highest DALYs were Centro (64.8), Obando (42.1) and Juanambú (39.1).

**Table 3:** Distribution of disability-adjusted life years (DALYs) in the department of Nariño, Colombia, 2020-2021.

Variables	Cases	Rates (per 1000 inhabitants)			RR DALY (crude)
		YLLD	YLD	DALY	
Period					
A	15,634	7.6	0.04	7.7	1.0
B	33,850	11.2	0.06	11.2	1.5
C	39,278	17.7	0.07	17.8	2.3
Age					
0 - 14	3580	1.0	0.03	1.0	1.0
15 - 29	21,594	4.6	0.15	4.8	4.6
30 - 44	29,477	27.1	0.26	27.4	26.6
45 - 59	20,353	74.3	0.27	74.6	72.4
60 - 69	7371	138.4	0.25	138.7	134.6
70 - 79	4054	177.3	0.21	177.5	172.3
80 or more	2333	189.8	0.22	190.0	184.5
Sex					
Female	46,417	26.8	0.18	27.0	1.0
Male	42,345	46.1	0.16	46.3	1.7
Ethnicity					
Indigenous	7278	28.0	0.13	28.1	1.0
Afrodescendant	5936	53.0	0.19	53.2	1.9
Other	75,548	36.3	0.17	36.4	1.3
Subregion					
Sanquianga	917	6.4	0.03	6.4	1.0
Centro	51,169	64.5	0.34	64.8	10.1
Guambuyaco	1371	21.5	0.11	21.6	3.4
Juanambú	4720	39.0	0.18	39.2	6.1
La Cordillera	1451	16.6	0.06	16.7	2.6
La Sabana	2553	34.0	0.13	34.1	5.3
Los Abades	903	8.5	0.03	8.5	1.3
Obando	12,668	42.0	0.16	42.2	6.6
Occidente	2183	31.0	0.14	31.1	4.8
Pacífico Sur	5232	26.5	0.1	26.6	4.1
Piedemonte Costero	405	27.6	0.05	27.7	4.3
Río Mayo	4153	18.8	0.11	18.9	2.9
Telembí	1037	11.4	0.05	11.5	1.8
Total	88,762	36.5	0.17	36.7	-

RR: rate ratios, YLLD: years lost due to premature death, YLD: years lived due to disability, DALY: disability-adjusted healthy life years.

A: March-August 2020 period, B: September 2020-February 2021 period, C: March-August 2021 period.

### Negative binomial regression model

We carried out the adjustment of the DALY rates by age in order to construct the model by using the direct method and taking the standard world population according to the World Health Organization (WHO) as reference.With this model we found that the variables age groups and subregion presented statistically significant associations (p < 0.05), and that the risk of DALYs is higher in age groups of 60 or more years than in those younger. The subregions with the highest risk were Piedemonte Costero (RR = 12.95), followed by Los Abades (RR = 2.54), which presented statistically significant differences with the reference subregion Sanquianga (p < 0.05), no statistical differences were found for the other subregions. No statistical differences were observed in the risks of DALYs by time period and sex (Table 4).

**Table 4:** Parameter estimation of the negative binomial regression model.

Variables	β	p-value	DALY RR (adjusted)	95% CI	
				LL	UL
(Intersection)	-7.832	0.000	0.000	0.000	0.001
Period					
A	0^a^		1		
B	0.241	0.321	1.273	0.790	2.050
C	0.426	0.071	1.532	0.964	2.435
Sex					
Female	0^a^		1		
Male	0.277	0.114	1.320	0.936	1.862
Age					
0 - 14	0^a^		1		
15 - 29	0.526	0.108	1.693	0.890	3.217
30 - 44	2.896	0.000	18.100	9.455	34.649
45 - 59	3.969	0.000	52.954	27.758	101.022
60 - 69	4.754	0.000	116.067	60.913	221.162
70 - 79	4.582	0.000	97.676	52.040	183.333
80 or more	4.263	0.000	71.035	37.826	133.402
Subregion					
Sanquianga	0^a^		1		
Centro	-0.522	0.215	0.593	0.260	1.354
Guambuyaco	0.398	0.354	1.490	0.641	3.459
Juanambú	0.147	0.732	1.159	0.499	2.691
La Cordillera	-0.471	0.273	0.625	0.269	1.448
La Sabana	0.041	0.924	1.042	0.448	2.425
Los Abades	0.931	0.041	2.538	1.040	6.197
Obando	-0.736	0.083	0.479	0.208	1.101
Occidente	0.268	0.531	1.307	0.566	3.015
Pacífico Sur	-0.156	0.710	0.856	0.377	1.946
Piedemonte Costero	2.561	0.000	12.946	5.469	30.642
Río Mayo	-0.630	0.159	0.533	0.222	1.280
Telembí	0.785	0.065	2.192	0.952	5.050
(scale)	3.661^b^	-	-	-	-
(Negative binomial)	1^c^	-	.-	-	-

a set to zero because this parameter is redundant; ^b^ calculated based on the deviation; ^c^ set to the displayed value.

RR: rate ratios, DALY: disability-adjusted healthy life years, LL: lower limit, UL: upper limit, β: beta coefficient.

A: March-August 2020 period, B: September 2020-February 2021, C: March-August 2021.

We calculated the determination coefficient, which measures the goodness of fit of the model to the data: D1 = log likelihood of the proposed model and D0 = log likelihood of the null model (only with the intercept).

Pseudo R^2^ = (D0 - D1) / D1 = 22.7%


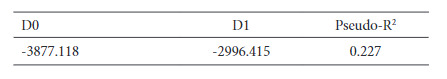


The variables period, age, sex, and subregion explain 22.7% of the variations in DALYs. 

Likewise, the size of the effect on the variations in the DALYs due to COVID-19 was determined from the four variables evaluated in the model, with the age group variable being the variable with the greatest weight, followed by the subregion variable and with a much smaller effect by period and sex, which explain globally about 23% of the variance of the DALYs.

## DISCUSSION

This is one of the first researches on COVID-19 disease burden worldwide. Although there are few studies on this subject, new results are published every day. In this research we observed that the burden of disease by COVID-19, for the department of Nariño, increased during the study period (it was significant between the first and third semester under observation), attributed to the increase in mortality, prior to the dissemination of vaccination. The most affected were men, those older than 70 years, population without ethnic affiliation, and those in subregions with a history of high incidence of chronic noncommunicable diseases.

The magnitude of the burden of disease found in this study relative to other types of flu (none pandemic) can be explained comparatively. For example, in 2022, WHO reported more than 300 million confirmed cases with more than 5 million deaths from COVID-19; whereas, for influenza, in 2022, WHO reported 1 billion cases, 3 to 5 million severe, which have resulted in 650,000 deaths. In other words, the lethality due to COVID-19, compared to other influenzas, is much higher and unprecedented as a public health problem ^9^.

Our results show that during the studied period (March 2020 - August 2021), the risk of morbidity due to COVID-19 in the department of Nariño increased significantly, the most affected were women, those over 45 years of age, people without ethnic affiliation, and those from the Central and Juanambú regions of the department of Nariño. The risk of death also increased during the study period, being higher in men, over 70 years of age, without ethnic affiliation; and the most affected regions were the Central and Juanambú regions. This agrees with a research carried out in Europe, which found that morbidity and lethality due to COVID-19 varies between regions, but that in countries such as Germany and Switzerland, the incidence by age and sex are higher in men aged 60 years and older ^23^.

It is important to note that the most populated subregions: Centro, Obando and Juanambú are the ones that occupy the first places in mortality due to COVID-19, but also according to the last study of disease burden ^20^ they are the ones that occupy the first places in mortality due to chronic diseases as well. This suggests a possible syndemic effect. According to a study in counties from the United States, there is a confluence of epidemics of both COVID-19 and chronic diseases in contexts of social disadvantage, where the African-American ethnic and population is the most affected group ^24^. A study based on data from 185 countries reports that COVID-19 morbidity and mortality correlate with the burden of chronic diseases, due to the aging of the population and the low capacity of health services to test and provide hospital beds, in socially unequal settings ^25^.

We found 97,950.8 DALYs with an annual rate of 24.5 per thousand inhabitants; and due to the recent and acute nature of the event, more than 97% of the burden (by age, sex, ethnicity, region, municipalities, among others), was contributed by the YLLD, which is explained because in the first two years of the pandemic, and prior to the introduction of vaccines ^26^, the people at greatest risk of death (men and those over 70 years of age), were the most affected by this event ^27^ ^-^ ^28^. This agrees with other studies, for example, one from the Netherlands reported that the DALY rate was 19.7 per 1000 inhabitants for age groups 85 years and older ^29^. Another study conducted in Ireland suggests that DALYs are significantly concentrated in populations aged 65 and over ^30^.

There are some similarities with a preliminary study on the burden of disease in the department of Córdoba (Colombia), which reports that 26.5 DALYs were lost per thousand inhabitants, and 99.9% of the burden of disease due to COVID-19 was due to YLLD; people who died from COVID-19 lost an average of 25 years of life, and 44% of the years of life lost corresponded to people under 60 years of age ^31^.

Similarly, a study in Germany reported that the percentage of DALYs in people under 70 years of age was 34.8% in men and 21.0% in women; people who died from COVID-19 lost an average of 9.6 years of life; those under 70 years of age who died lost an average of 25.2 years of life, more in men than in women ^32^. Also, a study in India found a rate loss of 10.2 DALYs per 1000 inhabitants and 14 million DALYs were reported due to the direct impact of COVID-19 in 2020 ^33^.

Gökler *et al*. ^34^, found that there is a significant difference between the average age of death and the average age of years of life lost (YLL) in a period of 1 year of pandemic in Turkey, reporting a difference of six years in men (average age of death: 69.79 years; average age of YLL: 63.67 years) and women (72.68; 66.07, respectively), premature deaths are included in the age group of 50 to 69 years. Likewise, it will be necessary to identify what happens with symptomatic cases; for the time being, in Colombia and in various countries of the world, the occupation of intensive care units has decreased considerably ^35^.

However, this event may become endemic due to vaccination ^36^; that is, that the number of cases reduces and/or stabilizes, where mortality decreases considerably, as has been observed in different scenarios worldwide ^37^ and most cases are asymptomatic, mild, or moderate ^38^. Regarding the aforementioned, a study carried out among healthcare workers shows that, among those vaccinated, 52.6% presented mild COVID-19 symptoms and 10.3% moderate symptoms, without serious illness or hospitalization ^39^.

In the future, mortality cases may occur mostly in the unvaccinated, as was the case at the end of 2021 in several European countries and in Asia ^40^, or may occur in those with known risk factors (advanced age, cardiovascular disease, metabolic and immune problems, among others) or unknown risk factors (immune or genetic) ^41^.

Our study has some limitations. We used the public database provided by the National Institute of Health which does not have clinical or sociodemographic variables different from those used in this research.The accuracy of the information provided by the source is variable and somewhat different from that presented by the official source of the National Statistics Department (this source is more accurate, but it may take 12 to 18 months to be completed due to the processes of analysis, review and source verification carried out by that entity). Overdispersion of the data forced the use of an alternative regression model to the one initially considered. Cases of persistent COVID-19 disease were not considered, a syndrome that has various forms of presentation, affecting both asymptomatic and hospitalized patients, with multiple symptoms, and requiring multidisciplinary management, which in many cases impacts quality of life. There may be an underestimation of the YDL morbidity component, due to possible underreporting and mild cases ^42^ ^,^ ^43^.

Despite the limitations, there are a number of advantages and potential benefits worth considering: this is one of the first studies at the regional level on the burden of disease in a given territory. Despite the potential source limitations, we used an official database to identify the magnitude and distribution of the problem, which is used by the national government. For the estimation of DALYs, we used microdata, i.e., contrary to what is done in other burden studies, which use aggregate information, in our study both YLLD and DALYs were estimated from the individual records of persons with morbidity or who died from this event, which is more precise. As an alternative, we used a negative binomial regression model to correct the overdispersion found in the data, which generates precise estimates of the risks adjusted for age, sex, region, and study period.

In conclusion, studies on the burden of disease due to COVID-19 during the pandemic, in regions and subregions, with the use of a standardized methodology such as that of Wyper *et al* ^22^ allow the estimation of DALYs. This study generates estimates that show that the population in Nariño with high indexes of multidimensional poverty are the most affected by COVID-19, which suggests that state resources should be focused in an intersectoral way in order to mitigate the effects on these populations, by means of social protection strategies and programs, through the management of policies that protect their livelihoods and promote human capital.
