# Supplementary material for: Burden of disease of COVID-19 in the department of Nariño, Colombia, 2020-2021
Source: Rev Peru Med Exp Salud Publica. 2022 Sep 30;39(3):281–91. doi: 10.17843/rpmesp.2022.393.10947 (PMC11397680; doi:10.17843/rpmesp.2022.393.10947)
Supplement: Translation

# Translation: Carga de enfermedad por COVID-19 en el departamento de Nariño, Colombia, 2020-2021

**Authors:** Arsenio Hidalgo-Troya, Jorge Martín Rodríguez, Anderson Rocha-Buelvas, Diana Urrego-Ricaurte

**Affiliations:** 1 Universidad de Nariño, Pasto-Nariño, Colombia.; 2 Pontificia Universidad Javeriana, Bogotá D.C., Colombia.

**Keywords:** SARS-CoV-2, COVID-19, Carga de Enfermedad, Años de vida Ajustados por la Incapacidad, Coronavirus, Colombia

## Abstract

**Objetivos.:**

Estimar la carga de enfermedad por COVID-19 en el departamento de Nariño, Colombia, a partir del indicador años de vida saludables ajustados por discapacidad (AVISA) entre marzo del 2020 a agosto del 2021.

**Materiales y métodos.:**

Con la información del sistema de vigilancia nacional SIVIGILA se hizo la descripción y caracterización de los casos de COVID-19 reportados entre marzo/2020 a agosto/2021, según grupos de edad, sexo, etnia, municipios de residencia y subregiones de Nariño. Se estimaron tasas crudas y de mortalidad acumulada por COVID-19 para las variables previamente descritas. Se calcularon años perdidos por muerte prematura (APMP) y años vividos por discapacidad (AVD). Se estimaron los AVISA totales mediante la suma de APMP + AVD. Estos se calcularon por sexo, etnia, grupo de edad y subregiones de Nariño. Se estimaron los riesgos relativos a partir de las razones de tasas (RT) e intervalos de confianza del 95% para las variables del estudio.

**Resultados.:**

Las tasas de morbilidad, mortalidad y AVISA más altas ocurrieron entre febrero y septiembre del 2021, en hombres, en mayores de 70 años, del grupo étnico minoritario afrodescendiente y en las subregiones Centro, Obando y Juanambú. La carga de enfermedad por COVID-19 en Nariño en el periodo de estudio es atribuida a los APMP, los cuales explican más del 97% de la misma.

**Conclusiones.:**

Este es uno de los primeros estudios de carga de enfermedad a nivel regional, realizados en Colombia, que emplea una metodología estandarizada para COVID-19. Esta medición generaría estimaciones que permitirían focalizar recursos de forma intersectorial, mitigar el daño a poblaciones y áreas geográficas determinadas, especialmente las más vulnerables.

## INTRODUCCIÓN

Los coronavirus se comportan como una zoonosis ^1^ ^) ^afectando la salud de los humanos en diferentes períodos. En los últimos 20 años ha generado varias epidemias. La primera fue el SARS (síndrome respiratorio agudo severo); entre 2002-2003 esta cepa infectó más de 8000 personas (China, Estados Unidos, Canadá, entre otros) produciendo una letalidad cercana a 10%. La segunda ocurrió en 2012, causando el MERS-CoV (síndrome respiratorio de medio oriente); esta epidemia afectó alrededor de 857 personas con una mortalidad cercana al 35% ^2^. El tercer brote fue producido por una nueva cepa (SARS-CoV-2), que apareció a finales de 2019 ^3^ causante de la emergencia sanitaria por la COVID-19, y que ha generado letalidades entre 1-10% a nivel mundial ^4^. La alta transmisibilidad se debe a la veloz diseminación respiratoria, debido a que la transmisión viral puede darse por pacientes sintomáticos o personas asintomáticas ^5^. 

Aunque el SARS-CoV-2 es una enfermedad parcialmente prevenible y tratable, para el mundo ha sido un desafío sanitario, económico, político y social. Para los países de bajos y medianos ingresos ha constituido la peor crisis humanitaria y de salud pública de su historia ^6^, con diferencias considerables en las tasas de morbilidad y mortalidad entre ellos ^7^. América Latina posee sistemas de salud con precarias capacidades para dar respuestas efectivas, manejan presupuestos en salud por debajo del 4,0% del producto interno bruto (PIB) ^8^ lo cual limita el acceso de calidad a la atención sanitaria, haciéndolos susceptibles de colapsar. Durante la pandemia, los gobiernos no asignaron recursos económicos y sociales eficientemente a las regiones más afectadas, lo cual generó profundización en las desigualdades económicas y sociales ^9^.

Existen otros determinantes sociales propios del continente que inciden en la salud poblacional. Por ejemplo, en Colombia, el 37% de los hogares no comen tres veces al día, y 43,9% de los jefes de hogar perciben que su economía ha empeorado ^10^. Colombia tiene la segunda tasa de desempleo más alta después de Haití, donde las mujeres son las más afectadas (39%) respecto a los hombres (18%). Asimismo, la informalidad laboral y la ampliación de la brecha de desigualdad social de Brasil y Colombia son muy altas ^11^, pero también esta brecha existe entre regiones de Colombia ^12^. Algunos de los determinantes anteriormente expuestos pudieron incidir en que a mediados de junio de 2021 el panorama epidemiológico por COVID-19 en Colombia fuera uno de los más desfavorable respecto al nivel mundial ^13^, afectando a personas con comorbilidades, las cuales requirieron atenciones especializadas ^14^. 

En Colombia el primer caso de COVID-19 se reportó en marzo/2020. En agosto/2021 el SARS-CoV-2 se había propagado a todo el país con cerca de cinco millones de casos confirmados y más de 122 000 muertes. A finales de 2020, el departamento de Nariño registró 88 927 casos confirmados y 3381 muertes, con una incidencia acumulada de 48/1000 habitantes y mortalidad de 1,8/1000 habitantes ^15^.

En países de bajos y medianos ingresos, la evaluación de la situación de salud poblacional se ha basado tradicionalmente en indicadores mortalidad como las tasas general o específica de mortalidad, mortalidad infantil, razón de mortalidad materna, entre otros; y/o de morbilidad como incidencias o prevalencias, dependiendo del tipo de evento objeto de estudio. Debido a sus limitaciones en la medición de fenómenos de salud-enfermedad, se ha generado la necesidad de desarrollar otros indicadores para medir aspectos relacionados como calidad de vida de las personas. En este contexto, los estudios de carga de enfermedad permiten identificar problemas de salud diversos causantes de muerte, morbilidad, y discapacidades, ya que integran la carga producida por muerte prematura, duración, secuelas de la enfermedad y discapacidad mediante el indicador años de vida saludables ajustados por discapacidad (AVISA) ^16^.

Colombia tiene antecedentes de varios estudios de carga de enfermedad, dentro de los cuales se encuentra los realizados por la Universidad Javeriana ^17^ ^,^ ^18^, y el Observatorio Nacional de Salud ^19^. El departamento de Nariño es el único en Colombia que cuenta con un estudio de carga de enfermedad por subregiones realizado por su autoridad sanitaria; en parte, debido a que es una región fronteriza caracterizada por poseer conjuntamente: contextos de conflicto armado, altos niveles de corrupción y territorios con población en y por debajo de la línea de pobreza ^12^, lo que aumenta el riesgo de mortalidad por COVID-19; de allí la relevancia de estudiar la situación del COVID-19 a partir de la estimación de los AVISA departamentales ^20^ y, en este caso, subregionales del departamento de Nariño.

En el escenario actual, es importante realizar estudios de carga de enfermedad no solo nacionales, sino también territoriales, ya que según lo indicado por el Departamento Administrativo Nacional de Estadística (DANE) y el Instituto Nacional de Salud de Colombia (INS), la COVID-19 es, actualmente, una de las más importantes causas de mortalidad y, por tanto, es esencial identificar cual es la carga atribuida a este evento, con el objeto de orientar acciones y políticas focalizadas ^21^. El objetivo del estudio fue determinar la carga de enfermedad atribuida a la COVID-19 en el departamento de Nariño, entre marzo del 2020 a agosto del 2021.

**MENSAJE CLAVE**

Motivación para realizar el estudio: los estudios de carga de enfermedad son estrategias metodológicas que aportan al análisis de situación de salud poblacional. Este es uno de los primeros estudios sobre la COVID-19 que estima los años de vida saludables ajustados por discapacidad (AVISA), adaptando una metodología a un contexto latinoamericano.

Principales hallazgos: la carga de enfermedad, al inicio de la epidemia para este territorio fue principalmente por mortalidad prematura, en hombres, mayores de 70 años, de grupos étnicos indeterminados y en territorios previamente afectados por enfermedades crónicas no transmisibles.

Implicancias: la estimación de AVISA orientan a los tomadores de decisiones a generar prioridades de intervención de recursos y de estrategias de control/prevención de esta enfermedad emergente a partir de evidencia científica.

## MATERIALES Y MÉTODOS

El presente es un estudio descriptivo analítico que emplea como fuente de información los registros de casos de COVID-19 notificados al Sistema de Vigilancia en Salud Pública Nacional (SIVIGILA) que coordina el INS. Para el presente estudio se tomaron los registros del departamento de Nariño.

A partir de la información del SIVIGILA se hizo la descripción y caracterización de los casos de COVID-19 reportados desde marzo/2020 a agosto/2021, por edades agrupadas, de la siguiente forma: (0-4, 5-14, 15-29, 30-44, 45-59, 60-69, 70-79 y 80 y +); sexo (hombres y mujeres); etnia (1: Indígena, 2:Gitanos, 3-5:Afroscendiente, 6:Otro ); municipios (todos los municipios del departamento de Nariño) y por las trece subregiones administrativas que maneja el departamento de Nariño (Obando, Occidente, Centro, Guambuyaco, Juanambú, La Cordillera, La Sabana, Los Abades, Pacífico Sur, Piedemonte Costero, Río Mayo, Sanquianga y Telembí). Para cada variable se obtuvo una descripción porcentual, y para el tiempo se dividió en tres semestres: marzo-agosto 2020 (A), septiembre-febrero de 2021 (B) y marzo-agosto de 2021 (C). 

### Carga de enfermedad de la COVID-19

Con base a las proyecciones de población del DANE 2020, se obtuvieron tasas crudas de incidencia de morbilidad (contagio) y mortalidad acumulada por COVID-19, para las variables previamente descritas (grupos de edad, sexo, etnia, municipio y subregión departamental). Se aplicaron pruebas de significancia estadística con un nivel de 0,05 para identificar cambios en las razones de tasas (RT) de AVISA por edad, sexo, subregión y periodo, mediante pruebas de Wald a través de un modelo lineal generalizado. 

Para la estimación de los AVISA se desagregó su cálculo tanto por los años perdidos por muerte prematura (APMP) como por años vividos por discapacidad (AVD) empleando algunos referentes metodológicos como se describe a continuación. Para el cálculo de los APMP se empleó la Tabla 1 que contiene las esperanzas de vida (EV) para ambos sexos, la cual toma como insumo principal las tasas menores de mortalidad registradas por cada grupo etario en países con más cinco millones de personas. La estimación se hizo multiplicando la EV por el volumen de muertes observadas en el mismo grupo etario, municipio, región, sexo, etnia específicos para el departamento de Nariño.

La ecuación para calcular los APMP, fue la siguiente:



Donde: Dx =1 al fallecer a X edad; Ex = esperanza de vida estándar de cada edad específica del modelo escogido y L = edad límite teórica de la tabla de vida elegida.

**Tabla 1:** Esperanzas de vida por grupos de edad utilizadas para el cálculo de años perdidos por muerte prematura (APMP).

Edad	EV	Edad	EV	Edad	EV	Edad	EV	Edad	EV
0	86,6	22	64,9	44	43,4	66	22,9	88	6,6
1	85,8	23	64,0	45	42,4	67	22,0	89	6,0
2	85,8	24	63,0	46	41,4	68	21,2	90	5,5
3	85,8	25	62,0	47	40,5	69	20,3	91	5,1
4	85,8	26	61,0	48	39,5	70	19,4	92	4,8
5	81,8	27	60,0	49	38,6	71	18,6	93	4,4
6	80,8	28	59,0	50	37,6	72	17,8	94	4,1
7	79,8	29	58,0	51	36,7	73	16,9	95	3,7
8	78,8	30	57,0	52	35,7	74	16,1	96	3,5
9	77,8	31	56,0	53	34,8	75	15,3	97	3,2
10	76,8	32	55,0	54	33,8	76	14,5	98	3,0
11	76,8	33	54,1	55	32,9	77	13,8	99	2,7
12	76,8	34	53,1	56	32,0	78	13,0	100	2,5
13	76,8	35	52,1	57	31,1	79	12,3	101	2,3
14	76,8	36	51,1	58	30,1	80	11,5	102	2,1
15	76,8	37	50,1	59	29,2	81	10,8	103	2,0
16	75,8	38	49,2	60	28,3	82	10,2	104	1,8
17	74,8	39	48,2	61	27,4	83	9,5	105	1,6
18	73,8	40	47,2	62	26,5	84	8,9	106	1,6
19	72,8	41	46,2	63	25,6	85	8,2	107	1,5
20	71,8	42	45,3	64	24,7	86	7,7	108	1,5
21	71,8	43	44,3	65	23,8	87	7,1	109-10	1,4

EV: esperanza de vida.

Fuente: Elaboración propia a partir del GBD Technical Training Workshop May 2015.

Para la estimación de los AVD, o años vividos con discapacidad para COVID-19, la fórmula empleada fue: *AVD*_*a*_ = D * L. Donde: a = la edad de comienzo de la enfermedad, D = peso de la enfermedad, el cual se ubica entre 0 y 1. Si el individuo es completamente sano se obtendría un D igual a 0, al contrario, si fallece D equivale a 1. Una enfermedad severa tiene un valor de D cercano a 1; un cuadro clínico leve tiene un valor de D aproximado a cero. Para la presente investigación, en cuanto al peso de la enfermedad, se usó la propuesta de Wyper *et al*. ^22^, donde se emplearon tres categorías: leve/moderado (0,051), severo (0,133) y crítico (0,655) (Figura 1). L=duración de la enfermedad, que para el presente caso se estimó, como fracción en años, con la diferencia entre fecha de recuperación y la fecha de inicio de síntomas según los registros del SIVIGILA.

**Figura 1:** Modelo de estimación de los años vividos por discapacidad para la COVID-1.

Los APMP y los AVD permitieron calcular los AVISA totales por medio de su sumatoria, de la siguiente forma: AVISA = APMP + AVD. De la misma forma, con base a las proyecciones de población del DANE se estimaron las tasas totales de AVISA por 1000 habitantes, por sexo, etnia, grupo de edad y por subregiones en el departamento de Nariño. 

### Modelo de regresión binomial negativo

Finalmente, se estimó un modelo lineal generalizado para identificar la tendencia de las tasas de los AVISA de COVID-19 para el periodo de estudio. Se calculó a partir del número de AVISA por COVID-19, las cuales se tomaron como una variable aleatoria, tomando Y = Ln(R), donde R representa las tasas de AVISA por COVID-19 y como variables independientes o explicativas a la edad como X1, a sexo como X2, al periodo de tiempo como X3, y a las regiones departamentales como X4. Se estimaron los riesgos relativos a partir de las razones de tasas (RT), junto con intervalos de confianza al 95%, para cada una de las categorías de las variables del estudio, a partir de unos grupos de referencia para cada una de las variables explicativas estudiadas. 

Para la estimación del modelo lineal generalizado se empleó el paquete estadístico IBM SPSS versión 23. Se verificaron los supuestos de equidispersión mediante los estadísticos de desvianza y chi cuadrado de Pearson para establecer si el modelo de regresión de Poisson era el modelo apropiado para los datos. Se identificó que los estadísticos de bondad de ajuste mostraron valores escala que indicaran sobredispersión, es decir, la violación a esta propiedad, por lo que se utilizó el modelo binomial negativo alternativo bajo esta situación, estimándose el estadístico pseudoR^2^ para establecer la calidad de ajuste del modelo. 

### Consideraciones éticas

Para la presente publicación, los datos analizados fueron extraídos de los registros notificados al sistema de vigilancia en salud pública nacional que coordina el INS. No se realizaron modificaciones de las cifras brindadas por las fuentes de información; cada vez que se hace referencia a un texto, se citan los autores, respetando los derechos de autor. Por lo anterior, según la resolución 8430 del año 1993 del Ministerio de Salud, esta investigación se clasifica sin riesgo, además este estudio cuenta con acta de Aprobación N.^o^ 004 del 23 de abril de 2020 del Comité de Ética en Investigación de la Vicerrectoría de Investigaciones e Interacción Social de la Universidad de Nariño.

## RESULTADOS

Durante el periodo marzo de 2020 y agosto de 2021 en el departamento de Nariño se presentaron 88 762 casos de contagio por la COVID 19, con una tasa anual de 3196 casos por cada cien mil habitantes. En el mismo periodo las muertes por el virus fueron igual a 2985 casos y una tasa anual de mortalidad de 107,5 casos por cada cien mil habitantes.

En la Tabla 2, en el componente de morbilidad, se observa que la tasa de incidencia acumulada (4794 por 100 000 habitantes) más alta ocurrió en los periodos B (1828,3) y C (2121,4). Esta tasa aumentó gradualmente con la edad, la más alta correspondió al grupo de 80 años y más (7779,8). Las mujeres tuvieron tasas de morbilidad levemente más altas (5034) que los hombres (4556,1). El grupo étnico minoritario más afectado fueron los afrodescendientes (4468,3). Las subregiones más afectadas fueron Centro (9955,3) y Juanambú (5400,6), superando el promedio departamental (4794,1). En el componente de mortalidad, también se observa que las tasas acumuladas más altas ocurrieron en los periodos B (56,87) y C (69,46). Las tasas de mortalidad (161,2 por 100 000 habitantes) también aumentaron gradualmente, siendo el grupo de 80 años y más el más afectado (2367,6). Al contrario de lo observado en la morbilidad, la mortalidad de hombres (199,3) fue mayor respecto a las mujeres (122,9). Nuevamente la etnia afrodescendiente tuvo una tasa de mortalidad más alta (211). Las subregiones con mayor mortalidad por COVID-19 fueron igualmente, Centro (293,6), Obando (180,3) y Juanambú (172,8).

**Tabla 2:** Morbilidad y mortalidad acumulada por la COVID-19 según características sociodemográficas en el departamento de Nariño, Colombia, 2020-2021.

Variables	Morbilidad			Mortalidad			Valor de p
	N	%	Tasas (x 100 mil)	N	%	Tasas (x 100 mil)	
Periodo de tiempo							< 0,001
A	15 634	17,6	844,4	646	21,6	34,9	
B	33 850	38,1	1.828,3	1053	35,3	56,9	
C	39 278	44,3	2121,4	1286	43,1	69,5	
Edad							< 0,001
0 - 4	829	0,9	492,9	4	0,1	2,4	
5 - 14	2751	3,1	846,1	2	0,1	0,6	
15 - 29	21 594	24,3	4557,4	35	1,2	7,4	
30 - 44	29 477	33,2	7488,4	221	7,4	56,1	
45 - 59	20 353	22,9	7337,0	595	19,9	214,5	
60 - 69	7371	8,3	6379,0	659	22,1	570,3	
70 - 79	4054	4,6	5982,8	759	25,4	1.120,1	
80 o más	2333	2,6	7779,8	710	23,8	2.367,6	
Sexo							< 0,001
Femenino	46 417	52,3	5034,0	1.133	38,0	122,9	
Masculino	42 345	47,7	4556,1	1.852	62,0	199,3	
Etnia							< 0,001
Indígena	7278	8,2	3525,2	251	8,4	121,6	
Afrodescendiente	5936	6,7	4468,2	280	9,4	210,8	
Otro	75 548	85,1	4996,0	2.454	82,2	162,3	
Subregión							< 0,001
Centro	51 169	57,6	9955,3	1509	50,6	293,6	
Guambuyaco	1371	1,5	3095,5	41	1,4	92,6	
Juanambú	4720	5,3	5400,6	151	5,1	172,8	
La Cordillera	1451	1,6	1754,3	62	2,1	74,9	
La Sabana	2553	2,9	3569,4	107	3,6	149,6	
Los Abades	903	1,0	941,5	35	1,2	36,5	
Obando	12 668	14,3	4124,2	554	18,6	180,4	
Occidente	2183	2,5	4358,9	77	2,6	153,7	
Pacífico Sur	5232	5,9	2189,8	250	8,4	104,6	
Piedemonte Costero	405	0,5	1455,0	32	1,1	114,9	
Río Mayo	4153	4,7	3731,4	101	3,4	90,7	
Sanquianga	917	1,0	728,6	31	1,0	24,6	
Telembí	1037	1,2	1097,3	35	1,2	37,0	
Total	88 762	100,0	4794,1	2.985	100,0	161,2	

Se calcularon los valores de p con el estadístico de chi cuadrado, que compara mortalidad y morbilidad acumulada.

A: periodo de marzo-agosto 2020, B: periodo de septiembre 2020-febrero 2021, C: periodo de marzo-agosto 2021. A: periodo de marzo-agosto 2020, B: periodo de septiembre 2020-febrero 2021, C: periodo de marzo-agosto 2021.

### Carga de enfermedad de la COVID-19

En el periodo analizado los APMP fueron 67 638,6 con una tasa anual de 25,3 por mil habitantes. Los AVD fueron 313,2 con una tasa anual de 0,11 por mil habitantes. Los AVISA en el periodo evaluado fueron 97 950,81 con una tasa anual de 24,5 por mil habitantes. En la Tabla 3 se observa que el tercer período de tiempo tuvo los mayores AVISA por COVID-19 (17,8). Por grupos de edad, se perdieron 190 AVISA en los de 80 años y más; 177,5 AVISAS en los de 70-79 años; 138,61 en los de 60-69 años y 74,55 en los de 45-59 años. Los hombres perdieron 46,3 AVISA, y las mujeres perdieron 27 por mil habitantes. El grupo étnico minoritario afrodescendiente perdió 53,2 AVISA. Finalmente, las subregiones con más AVISA fueron Centro (64,8), Obando (42,1) y Juanambú (39,1).

**Tabla 3:** Distribución de de los años de vida saludables ajustados por discapacidad (AVISA) en el departamento de Nariño, Colombia, 2020-2021.

Variables	Casos	Tasas (x mil habitantes)			RT AVISA (crudas)
		APMP	AVD	AVISA	
Periodo					
A	15 634	7,6	0,04	7,7	1,0
B	33 850	11,2	0,06	11,2	1,5
C	39 278	17,7	0,07	17,8	2,3
Edad					
0 - 14	3 580	1,0	0,03	1,0	1,0
15 - 29	21 594	4,6	0,15	4,8	4,6
30 - 44	29 477	27,1	0,26	27,4	26,6
45 - 59	20 353	74,3	0,27	74,6	72,4
60 - 69	7 371	138,4	0,25	138,7	134,6
70 - 79	4 054	177,3	0,21	177,5	172,3
80 o más	2 333	189,8	0,22	190,0	184,5
Sexo					
Femenino	46 417	26,8	0,18	27,0	1,0
Masculino	42 345	46,1	0,16	46,3	1,7
Etnia					
Indígena	7 278	28,0	0,13	28,1	1,0
Afro descendiente	5 936	53,0	0,19	53,2	1,9
Otro	75 548	36,3	0,17	36,4	1,3
Subregión					
Sanquianga	917	6,4	0,03	6,4	1,0
Centro	51 169	64,5	0,34	64,8	10,1
Guambuyaco	1 371	21,5	0,11	21,6	3,4
Juanambú	4 720	39,0	0,18	39,2	6,1
La Cordillera	1 451	16,6	0,06	16,7	2,6
La Sabana	2 553	34,0	0,13	34,1	5,3
Los Abades	903	8,5	0,03	8,5	1,3
Obando	12 668	42,0	0,16	42,2	6,6
Occidente	2 183	31,0	0,14	31,1	4,8
Pacífico Sur	5 232	26,5	0,1	26,6	4,1
Piedemonte Costero	405	27,6	0,05	27,7	4,3
Río Mayo	4 153	18,8	0,11	18,9	2,9
Telembí	1 037	11,4	0,05	11,5	1,8
Total	88 762	36,5	0,17	36,7	-

RT: razones de tasas, APMP: años perdidos por muerte prematura, AVD: años vividos por discapacidad, AVISAS: años de vida saludables ajustados por discapacidad.

A: periodo de marzo-agosto 2020, B: periodo de septiembre 2020-febrero 2021, C: periodo de marzo-agosto 2021.

### Modelo de regresión binomial negativo

Para la construcción del modelo se realizó el ajuste de las tasas de AVISA por edad, utilizando el método directo y tomando como referencia la población mundial estándar según la Organización Mundial de la Salud (OMS). Este modelo permitió observar que las variables grupos de edad y subregión presentaban asociaciones estadísticamente significativas (p < 0,05), encontrando que el riesgo de AVISA es mayor en los grupos de edad de 60 o más años que en los de edades inferiores. Las subregiones de mayor riesgo fue Piedemonte Costero (RT = 12,95), seguida de Los Abades (RT = 2,54), que presentaron diferencias estadísticamente significativas con la subregión de referencia Sanquianga (p < 0,05), no se observaron diferencias estadísticas en las demás subregiones. Por periodo de tiempo y sexo no se observaron diferencias estadísticas en los riesgos de AVISA (Tabla 4).

**Tabla 4:** Estimación de parámetros del modelo de regresión binomial negativo.

Variables	β	Valor de p	RT AVISA (ajustadas)	IC 95%	
				LI	LS
(Intersección)	-7,832	0,000	0,000	0,000	0,001
Periodo					
A	0^a^		1		
B	0,241	0,321	1,273	0,790	2,050
C	0,426	0,071	1,532	0,964	2,435
Sexo					
Femenino	0^a^		1		
Masculino	0,277	0,114	1,320	0,936	1,862
Edad					
0 - 14	0^a^		1		
15 - 29	0,526	0,108	1,693	0,890	3,217
30 - 44	2,896	0,000	18,100	9,455	34,649
45 - 59	3,969	0,000	52,954	27,758	101,022
60 - 69	4,754	0,000	116,067	60,913	221,162
70 - 79	4,582	0,000	97,676	52,040	183,333
80 o más	4,263	0,000	71,035	37,826	133,402
Subregión					
Sanquianga	0^a^		1		
Centro	-0,522	0,215	0,593	0,260	1,354
Guambuyaco	0,398	0,354	1,490	0,641	3,459
Juanambú	0,147	0,732	1,159	0,499	2,691
La Cordillera	-0,471	0,273	0,625	0,269	1,448
La Sabana	0,041	0,924	1,042	0,448	2,425
Los Abades	0,931	0,041	2,538	1,040	6,197
Obando	-0,736	0,083	0,479	0,208	1,101
Occidente	0,268	0,531	1,307	0,566	3,015
Pacífico Sur	-0,156	0,710	0,856	0,377	1,946
Piedemonte Costero	2,561	0,000	12,946	5,469	30,642
Río Mayo	-0,630	0,159	0,533	0,222	1,280
Telembí	0,785	0,065	2,192	0,952	5,050
(Escala)	3,661^b^	-	-	-	-
(Binomial negativa)	1^c^	-	.-	-	-

a Definido en cero porque este parámetro es redundante; ^b^ se calcula basándose en la desvianza; ^c^ fijado en el valor visualizado.

RT: razones de tasas, AVISA: años de vida saludables ajustados por discapacidad, LI: limite inferior, LS: limite superior, β: coeficiente beta.

A: periodo de marzo-agosto 2020, B: periodo de septiembre 2020-febrero 2021, C: periodo de marzo-agosto 2021.

Se calculó el coeficiente de determinación, que mide la bondad de ajuste del modelo a los datos: D1 = log verosimilitud del modelo propuesto y D0 = log verosimilitud del modelo nulo (solo con el intercepto).

Pseudo R^2^ = (D0 - D1) / D1 = 22,7%



Las variables periodo, edad, sexo y subregión explican en un 22,7% las variaciones de los AVISA. 

Igualmente, se determinó el tamaño del efecto en las variaciones de los AVISA de la COVID-19 a partir de las cuatro variables evaluadas en el modelo, siendo la variable grupos de edad la variable de mayor peso, seguida por la variable subregión y con mucho menor efecto período y sexo, las cuales explican de manera global cerca de un 23% de la varianza de los AVISA.

## DISCUSIÓN

Esta es una de las primeras investigaciones de carga de enfermedad por COVID-19 a nivel mundial. Aunque son escasos los estudios publicados en relación con este tema, día a día se conocen nuevos resultados de investigaciones relacionadas. En esta investigación se observó que la carga de enfermedad por la COVID-19, para el departamento de Nariño, se incrementó durante el período de estudio (fue significativo entre el primer y tercer semestre objeto de observación), atribuido al incremento de la mortalidad, previo a la diseminación de la vacunación; los más afectados fueron los hombres, mayores de 70 años, población sin pertenencia étnica, y en subregiones con antecedentes de alta incidencia de enfermedades crónicas no transmisibles. 

La magnitud de la carga de enfermedad encontrada en este estudio respecto a otras gripes (ninguna pandémica), puede ser explicada comparativamente. Por ejemplo, en 2022, la OMS reportó más de 300 millones de casos confirmados con más de cinco millones de muertes por COVID-19; mientras que, para influenza, en 2022, reportó 1000 millones de casos, 3 a 5 millones graves, los cuales han producido 650 000 muertes. Es decir, la letalidad por COVID-19, respecto a otras gripes, es mucho mayor y sin precedentes como problema de salud pública ^9^.

Del presente trabajo de investigación se resalta que, durante el período estudiado (marzo 2020 -agosto 2021), el riesgo de morbilidad por COVID-19 en el departamento de Nariño se incrementó significativamente, los más afectados fueron las mujeres, los mayores de 45 años, las personas sin pertenencia étnica, de las regiones Centro y Juanambú del departamento de Nariño. En cuanto al riesgo de muerte, este también se incrementó en el período de estudio, siendo mayor en hombres, mayores de 70 años, sin pertenencia étnica, y las regiones más afectadas fueron la Centro y Juanambú. Lo anterior concuerda con una investigación realizada en Europa, donde se observó que la morbilidad y letalidad por COVID-19 varía entre regiones, pero que, en países como Alemania y Suiza, la incidencia por edad y sexo son mayores en hombres de 60 y más años ^23^. 

Resulta de interés que en las subregiones más pobladas: Centro, Obando y Juanambú son las que ocupan los primeros lugares en mortalidad por COVID-19, pero también según el último estudio de carga de enfermedad ^20^ son las que ocupan los primeros lugares en mortalidad por enfermedades crónicas. Lo anterior sugiere un posible efecto sindémico; según un estudio en condados de los Estados Unidos existe una confluencia de epidemias, tanto de la COVID-19 como de enfermedades crónicas en los contextos de desventaja social, donde el grupo étnico y poblacional afroamericano son mayoría ^24^; y según un estudio basado en datos de 185 países reporta que la morbilidad y mortalidad por la COVID-19 se correlacionan con la carga de enfermedades crónicas, esto debido al envejecimiento de la población y a la baja capacidad de los servicios de salud para realizar pruebas y proporcionar camas hospitalarias, desde luego, en contextos socialmente desiguales ^25^. 

En cuanto a los AVISA, fueron 97 950,8 con una tasa anual de 24,5 por mil habitantes; por lo reciente y agudo del evento, más del 97% de la carga (por edad, sexo, etnia, región, municipios, entre otros), fue aportada por los APMP, lo cual se explica porque en los dos primeros años de la pandemia, y previo a la introducción de las vacunas ^26^, las personas con mayor riesgo de muerte (hombres y mayores de 70 años), eran los más afectados por este evento ^27^ ^-^ ^28^. Lo anterior coincide con otras investigaciones, por ejemplo, una de los países bajos resaltó que la tasa de AVISA fue de 19,7 por mil habitantes para los grupos de edad de 85 y más años ^29^. Otro estudio realizado en Irlanda sugiere que los AVISA se concentran significativamente en las poblaciones de 65 y más años ^30^. 

También existe ciertas coincidencias con un estudio preliminar de carga de enfermedad hecho en el departamento de Córdoba (Colombia), el cual reporta que se perdieron 26,5 AVISA por mil habitantes, donde el 99,9% de la carga de enfermedad por la COVID-19 se debió a APMP, las personas fallecidas por la COVID-19 perdieron en promedio 25 años de vida, y el 44% de los años de vida perdidos corresponden a personas menores de 60 años ^31^.

En ese mismo sentido, en Alemania se reportó que el porcentaje de AVISA en menores de 70 años fue 34,8% en hombres y 21,0% en mujeres, las personas que fallecieron por la COVID-19 perdieron una media de 9,6 años de vida; los menores de 70 años que fallecieron perdieron una media de 25,2 años de vida, más en hombres que en mujeres ^32^. También, en la India, se reportó una pérdida de tasa de 10,2 AVISA por mil habitantes y 14 millones AVISA debido al impacto directo de la COVID-19 en 2020 ^33^. 

Gökler *et al*. ^34^, encontraron que existe una diferencia significativa entre la edad promedio de muerte y la edad promedio de años de vida perdidos (AVP) en un período de 1 año de pandemia en Turquía, reportando una diferencia de seis años en hombres (edad media de muerte: 69,79 años; edad media de AVP: 63,67 años) y mujeres (72,68; 66,07, respectivamente), las muertes prematuras se recogen en el grupo de edad de 50 a 69 años. Igualmente, habrá que identificar qué pasa con los casos sintomáticos; por ahora, en Colombia y en diversos países del mundo, la ocupación de las unidades de cuidados intensivos ha disminuido considerablemente ^35^.

Ahora bien, es posible que con la vacunación este evento pase a ser una endemia ^36^; es decir, que el número de casos se reduzca y/o estabilice, donde la mortalidad disminuya considerablemente, como se ha observado en diferentes escenarios a nivel mundial ^37^ y predominen casos con el espectro observado al inicio de la pandemia, principalmente con casos asintomáticos, leves o moderados ^38^. En relación con lo anterior, una investigación realizada en trabajadores de la salud muestra que, entre los vacunados, el 52,6% presentaron cuadros de COVID-19 leve, y 10,3% cuadro moderado, sin enfermedad grave ni hospitalización ^39^.

En el futuro, los casos de mortalidad que ocurran es posible que se concentren en los no vacunados, como está ocurriendo a finales de 2021 en diversos países europeos y en Asia ^40^, o que ocurran en aquellas personas con factores de riesgo conocidos (edad avanzada, enfermedad cardiovascular, problemas metabólicos, inmunes, entre otros) o no conocidos (inmunes o genéticos) ^41^. 

El presente estudio tiene algunas potenciales limitaciones: el acceso a la información fue posible por la base pública dispuesta por el Instituto Nacional de Salud, la cual no cuenta con variables clínicas, ni sociodemográficas diferentes a las usadas en la presente investigación. La precisión de la información que aporta la fuente es variable y un poco diferente a la presentada por la fuente oficial del Departamento Nacional de Estadística (esta fuente es más precisa, pero también se sabe que puede tardar 12 o 18 meses en ser completada debido a los procesos de análisis, crítica y verificación de fuente que realiza dicha entidad). Los datos tenían una sobredispersión, lo cual obligó al empleo de un modelo de regresión alternativo al originalmente considerado. No se tuvieron en cuenta los casos de enfermedad de COVID-19 persistente, síndrome que tiene diversas maneras de presentación, y que afecta tanto pacientes asintomáticos como a hospitalizados, con múltiples sintomatologías, y que requieren un manejo multidisciplinario, lo cual impacta, en muchos casos, la calidad de vida. Puede haber una subestimación del componente de morbilidad AVD, a partir del posible subreporte y de casos leves ^42^ ^,^ ^43^.

A pesar de las anteriores limitaciones, hay una serie de ventajas y potenciales beneficios que vale la pena considerar: este es uno de los primeros estudios a nivel regional sobre la carga de enfermedad en un territorio determinado. A pesar de las potenciales limitantes de la fuente, se empleó una base de datos también oficial que genera oportunidad en la información, es la empleada por el gobierno nacional para identificar la magnitud y distribución del problema. Para la estimación de los AVISA se emplearon los microdatos, es decir, al contrario, como se hace en otros estudios de carga, donde se usa información agregada, en el presente estudio se estimaron tanto los APMP y los AVD a partir de los registros individuales de personas con morbilidad o que fallecieron por este evento, lo que genera gran precisión en la información arrojada. Se empleó como alternativa un modelo de regresión binomial negativo, que corrigió la sobredispersión observada en los datos, lo cual genera estimaciones precisas en los riesgos ajustados por edad, sexo, región y período de estudio. 

En conclusión, los estudios de carga de enfermedad por COVID-19 durante la pandemia, en regiones y subregiones, con la utilización de una metodología estandarizada como la de Wyper *et al.* ^22^ que permite estimar AVISA. Este estudio genera estimaciones que indican que la población más afectada por la COVID-19 en Nariño es aquella con altos índices de pobreza multidimensional, lo que sugiere que se deben focalizar recursos estatales de forma intersectorial para mitigar los efectos en dichas poblaciones, por medio de estrategias y programas de protección social, a través de la gestión de políticas que protejan sus medios de sustento y promuevan el capital humano.
